# Preparation and Characterization of Novel Oleogels Using Jasmine Floral Wax and Wheat Germ Oil for Oral Delivery of Curcumin

**DOI:** 10.1021/acsomega.2c03201

**Published:** 2022-08-17

**Authors:** Anashwara Babu, Gomathi Sivakumar, Anubhab Das, Deepti Bharti, Dilshad Qureshi, SK Habibullah, Anjana Satheesan, Biswaranjan Mohanty, Kunal Pal, Samarendra Maji

**Affiliations:** † Department of Chemistry, SRM Institute of Science and Technology, Kattankulathur, Chennai 603203, India; ‡ Department of Biotechnology and Medical Engineering, National Institute of Technology Rourkela, Rourkela, Odisha 769008, India; § Institute of Pharmacy and Technology, Salipur, Odisha 754202, India

## Abstract

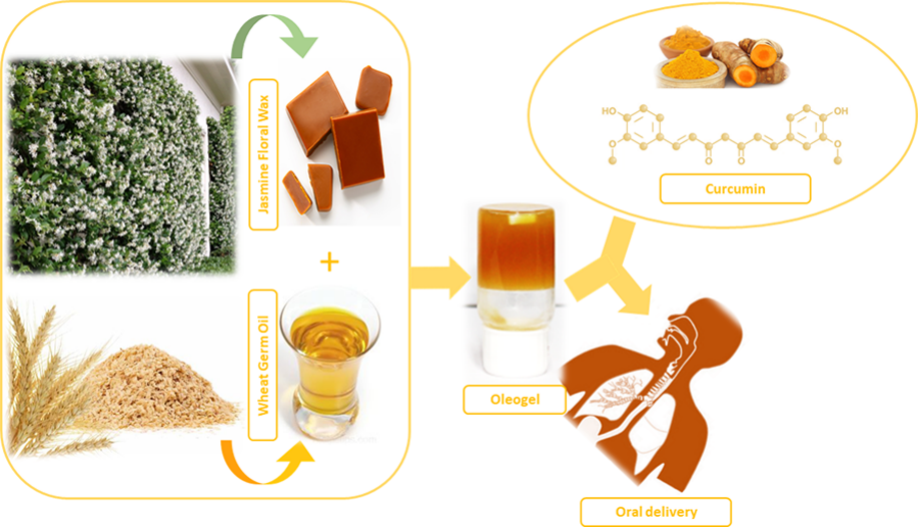

Oleogels (OGs) have gained a lot of interest as a delivery system for a variety of pharmaceuticals. The current study explains the development of jasmine floral wax (JFW) and wheat germ oil (WGO)-based OGs for oral drug (curcumin) delivery application. The OGs were made by dissolving JFW in WGO at 70 °C and cooling it to room temperature (25 °C). The critical gelation concentration of JFW that induces the gelation of WGO was found to be 10% (w/w). The OGs were characterized using various techniques such as Fourier transform infrared spectroscopy (FTIR), X-ray diffraction (XRD), microscopic analysis, and mechanical test. XRD data indicated that JFW influences the crystallinity of the OGs. Among the prepared OGs, OG 17.5 showed higher crystallization in the series. Optical microscopic studies demonstrated the formation of fiber structures due to the entanglement of crystals whereas, polarized light micrographs suggested the formation of spherulites or clustered crystallite structures. The mechanical properties of the OGs increased linearly with the increase in the JFW concentration. Curcumin-loaded OGs were examined for their controlled release applications. In summary, the developed OGs were found to have the necessary features for modulating the oral delivery of curcumin.

## Introduction

Semisolid systems made by the immobilization of organic oils into a three-dimensional polymer network structure using gelator molecules are called oleogels (OGs).^1^ The wide variety of applications of OGs in the food industry to substitute trans- and saturated fats in food items including baking, dairy, and meat products, as well as to serve in biomedical and cosmetics fields enhance their importance among researchers.^2^ Oleogelators are solid components used to arrest the motion of an edible oil resulting in OGs.^3^ Oil structuring/oleogelation is the method of providing solid properties to oil and this process takes place owing to the gelation of oil-induced by an oleogelator. The critical gelation concentration (CGC) is the minimum concentration of the gelator molecule to induce gelation. The gel-based systems exhibit solid or solid-like behavior even though the proportion of the liquid fraction is higher than the solid fraction in the system. This self-assembly is happening via hydrogen bonding, van der Waals forces, ionic interactions, and π–π stacking between oil and gelator molecules.^4^ In general, two different types of gelators, such as low-molecular-weight oleogelators (LMGOs; e.g., fatty acids) and HMGOs (high-molecular-weight oleogelators; e.g., polysaccharides), are used to form structured OG networks. Many edible oils like wheat germ oil (WGO), safflower oil, flaxseed oil, olive oil, sesame oil, grapeseed oil, soybean oil, algal oil, palm oil, salmon oil, and combinations are most commonly used in OG preparations.^5^ From the oils mentioned above, WGO is vibrantly suitable in developing healthy, safe, and nutritive OGs.^5^ Wheat germ is a by-product of wheat milling that can produce up to 10% oil, which can be separated mechanically or chemically. Alternative techniques such as supercritical fluid fractionation and molecular distillation can be used instead of conventional refining to improve the nutritional quality of the oil. WGO is high in nutrients because it is high in omega-3, omega-6, and tocopherols (especially vitamin E), which help in the regulation of the nervous system.^6^ WGO consist of an alcohol named octacosanol, which can reduce cholesterol by promoting physical performance including stamina and strength. It helps in bringing down LDL cholesterol and also prevents hardening of arteries and enhances cardiovascular health. WGO’s color is largely determined by carotenoids and flavonoid glycosides. WGO is used in the fields of medicine, cosmetics, agriculture, and food.^7^ Jasmine floral wax (JFW) having *Jasminum grandiflorum* as the scientific name is an aromatic by-product derived from manufacturing jasmine absolute.^8^ They are used in traditional medicines because of their antiseptic, antimicrobial, relaxant, thermogenic, and tonic properties.^9^ Solid perfumes, balms, lotions, and other solid or semisolid products benefit from the aromatic addition of JFW. It is an excellent vegan substitute for bee wax in recipes that call for it. It is slightly softer than bee wax, and some trial and error may be required.^8^

Over many years, researchers have been working on new ways to prepare OGs for use as a substituent in the food industry. Martins et al. explain the gelation mechanism and excellent quality structurants utilized to make OGs.^10^ Later, Puscas et al. emphasized in a review the negative consequences created by unhealthy and controversial fat in food, emphasizing the significance of replacing them with palatable OGs.^11^ They have also reviewed the range of OGs synthesized and their use in all conceivable food. Methods to circumvent the difficulty of using OGs instead of harmful fatty acids in practice are also detailed in a recent review.^12^ This offers a notion of the desire to use OGs on a budget without sacrificing food quality.

OGs made from sunflower oil or high oleic sunflower oil with glyceryl monostearate as an oleogelator^13^ and soybean oil mixed with rice bran wax^14^ were successfully invented and experimentally proven as a healthier, sensorily-acceptable substitute for saturated fat in bologna sausage. Similarly, utilizing a composite mixture of whey protein, potato starch, and beeswax-based OG in sunflower oil, a highly advanced 3D printing technique currently widely used in the field of food was used to study the accuracy, customization, and modification in food. The 30% OG content combination was optimal for 3D printing, and the OG generated in the study served as a reference for high oil systems in 3D printing.^15^ Another study looked at the influence of a monoglyceride gelator, such as glycerol monolaurate, on the physical and oxidative stability of OG made from camellia oil, which may be utilized in food formulation.^16^ When sesame oil OGs formulated with 10% ethylcellulose were used to substitute animal fat in beef burgers, the oxidation process was slowed, fat absorption was decreased, the cooking loss was minimized, and nutritional, quality, and chewiness were improved.^17^ Nagavekar et al., extracted kokum fat and used it for OG preparation with oils.^18^ The rheological profile, thermal stability, crystallinity, and Fourier transform infrared (FTIR) spectroscopy properties of the prepared OGs were all evaluated. They show vast and efficient application in the food industry to decrease saturated fatty acid use with no change in the quality of product and taste. Thus, several OGs with oil and wax combinations are already well-known in this field, and recent improvements are also extremely rapid. Other OGs show promising application in drug delivery applications as well. Semisolid systems like OGs act as hydrophilic excipients, as it includes mainly lipophilic excipients. Thus, OGs promote the penetration of hydrophobic drugs via the lipophilic pathway. Many organic substances like lipids present in OGs enhance their activity as a hydrophobic drug carrier.^19^ In particular, vegetable oil-based OGs are easy to prepare, inexpensive, biocompatible, nontoxic, and can incorporate both hydrophobic and hydrophilic drugs.^20,21^ Additional advantages like long shelf life and resistance to microbial contamination in some cases are also proven.^22^ Because of their superior viscosity and spreadability, OGs have been successfully studied as dermal pharmaceuticals, outperforming hydrogels and microemulsions.^23,24^

The oral bioavailability of a drug relies on the aqueous solubility and intestinal permeability of the drug.^25^ Curcumin’s low water solubility leading to poor systemic absorption and low bioavailability due to rapid metabolism are two key drawbacks of utilizing it in traditional dosage techniques.^26^ Curcumin degrades under aqueous conditions at both neutral and alkaline pH. Due to its antioxidant, anti-inflammatory, and anticancer properties, Curcumin (diferuloylmethane), a lipophilic polyphenol substance extracted from the herb’s rhizomes, has been playing a significant therapeutic role in a variety of diseases, including diabetes, inflammatory disorders, and various types of cancers.^27^ Mahmood et al. have written a comprehensive review of curcumin’s antibacterial characteristics as well as its vast range of applications in the medical field.^28^ A recent work by Li et al. revealed curcumin-loaded OGs built using β-sitosterol and lecithin with physical properties and in vitro curcumin release behavior.^29^ The oxidation stability of the delivery system which was determined by an accelerated oxidation test proved that the oxidative stability of curcumin-loaded OG was higher than that of free OG and corn oil-curcumin mixtures. Up to 67.66% of enhancement of bioaccessibility of curcumin was confirmed through in vitro analysis. A 3D printed medium-chain triglycerides oleogel used for the delivery of curcumin and resveratrol was developed recently which helps in the personalized delivery of drugs according to the need of patients.^30^ A similar study was done on curcumin release by Palla et al. in which curcumin was loaded within stable nanoemulsions made from monoglyceride OGs.^31^ The preparation of OGs with single or multiple oleogelators, as well as their uses in providing nutrients or substituting solid fats with OGs, are discussed in a recent review.^32^ This article also discusses some of the significant challenges that come with integrating them into commercial goods.

In our research, we employed WGO as the oil phase and JFW as the gelator, and we looked into their potential applications in the food industry as both the oleogelator (wax) and oil are edible and plant-based. Interestingly, no one has looked into the process of making OG from WGO and JFW till now. The OG may be made by dissolving various concentrations of JFW in WGO at a temperature over the melting point of JFW, which is roughly 65 °C, and then cooling to room temperature. Since this is the first OG made from WGO utilizing JFW, the CGC of JFW, or the minimal concentration of JFW necessary for WGO gelation, was calculated with deliberate effort. After finding the CGC, OGs with various concentrations of JFW ranging from 10 to 20% (w/w) were prepared. These OGs were subjected to characterization such as X-ray diffraction (XRD), IR spectroscopy, microstructural, crystallization, and metallurgical studies to confirm the OG formation. Furthermore, in vitro and ex vivo drug release tests were also used to assess the release of curcumin infused in the produced OGs. Thus, studies are done with prepared OGs to prove their applicability in the food industry, specifically for oral drug delivery.

## Experimental Section

### Materials

WGO, 100% pure and natural, was procured from AOS Products Private Limited, Ghaziabad, India, and JFW was purchased from Vijay Impex Pvt. Ltd., India. Curcumin was purchased from Himedia Laboratories Pvt. Ltd., Mumbai, India (MW 368.39). Disodium hydrogen phosphate and potassium dihydrogen phosphate were purchased from Rankem Pvt. Ltd., Haryana, India. Sodium lauryl sulfate was purchased from SRL Pvt. Ltd., Mumbai, India.

### Preparation of the Oleogel

WGO was used as the base oil and JFW as the oleogelator in the preparation of OGs. To identify the CGC, a series of OGs in 10 mL clean glass vials were prepared by varying the quantities of JFW and WGO. The concentration of the JFW was set at six different concentrations, 5, 8, 10, 12, 15, and 25% w/w. Later, these sample mixtures were heated in a water bath at 70 °C to dissolve the JFW within the WGO. The melted solutions were homogenized using a magnetic stirrer for 10 min at the aforesaid temperature. Subsequently, these homogenized solutions were kept at room temperature (∼28 °C) overnight to induce the crystallization of gelator molecules.

Following the confirmation of the CGC, a series of OGs were made in 10 mL glass vials to evaluate the influence of gelator molecules on the characteristics of OGs. The JFW content in the OGs varied between 10%, 12.5, 15, 17.5, and 20 w/w, and the OGs were prepared using the same process. The samples were named OG 10, OG 12.5, OG 15, OG 17.5, and OG 20, respectively.

### Study of Oil Released and Oil Binding Capacity

For analyzing oil release and oil binding capacity (OBC), the procedure by Thakur et al. was used.^33^ Prepared OG samples were melted and transferred to previously weighed Eppendorf. After gelation of the sample, Eppendorf with OG was weighed and stored in a refrigerator for 24 h. Then the samples were centrifuged for 15 min at 10,000 rpm. After centrifugation, samples were inverted and the oil excreted was drained and wiped. The Eppendorf was weighed again. The calculation for % OBC can be done using eqs 1 and 2.
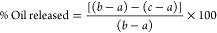
1
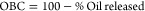
2

### Colorimetric Analysis

A colorimeter developed in the laboratory was used to analyze the OGs. The colorimeter device’s hardware comprises a light-emitting diode (LED) as a light source and Picam for imaging purposes.^34^ Before the experiment, the equipment was standardized using the conventional white and black tiles. The experiment was carried out using OGs placed in 35 mm Petri-dishes. Following that, the values of the color coordinates L*, a*, and b* were determined. The photos of the samples were then captured to determine several color characteristics such as L* (lightness), a* (+*a* redness and *a* greenness), and b* (+*b* yellowness and *b* blueness). The yellowness index (YI) was derived using the color parameter values mentioned earlier. The following formula, as given in eq 3, was utilized for this purpose.
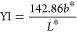
3

### Molecular Characterization

#### FTIR Spectroscopy

The IR spectra of all the OGs were examined using an FTIR spectrophotometer (Alpha-E; Bruker, Billerica, MA, USA) in the attenuated total reflectance (ATR) mode. The OGs were scanned with 25 scans each in the wavenumber range 600–4000 cm^–1^. The spectral resolution of the instrument was 4 cm^–1^. The same method was used to obtain the IR spectra of pure JFW and WGO.

### X-Ray Diffraction

A diffractometer of Bruker’s ″DAVINCI design″, the D8 ADVANCE system, was used to capture wide-angle X-ray diffractograms. A copper source was used to generate X-rays (Cu-Kα radiation; 1.5604 Å). The sample was scanned in the range of 5-80^o^ 2θ with a continuous scanning rate of 5° 2θ/min.

### Crystallization Studies

The gelation kinetics study was conducted using a colorimeter (CL223 Colorimeter, Elico Ltd., Hyderabad, India). The OG was heated to 70 °C and filled in the sample holder of the colorimeter. The optical density (OD) and the temperature were recorded until the temperature of the OG reached 25 °C. The time and the OD were also recorded. The graph of OD versus temperature and OD versus time was plotted.

### Microscopic Analysis

An upright bright-field compound microscope (Leica Microsystems, model: DM750, GmbH, Wetzlar, Germany) with an in-house developed polarizer was used to visualize the optical microscopy of the prepared OGs. The micrographs were seen in both bright-field and polarized modes. The molten OGs drop was put on a glass slide and covered with a coverslip before being analyzed.

### Metallurgical Studies

This study was done with the help of a metallurgical microscope (AmScope, ME580TA, Irvine, CA, USA). For this purpose, ∼5.0 g of molten OG sample was placed in a 35 mm petri-dish where it was allowed to solidify. The solidified OGs were then used for surface analysis under the microscope.

### Mechanical Study

#### Stress Relaxation

Stress relaxation (SR) tests were used to investigate the mechanical characteristics of the OGs. The texture analyzer HD plus tool was used in the investigation (Godalming, Surrey, UK). The OGs were made in 100 mL polypropylene beakers for the SR study. The trigger force was initially set to 5.0 g. An acrylic male conical probe (angle 45°) was used to pierce the solidified OG with a continuous strain of 1 mm at a rate of 0.5 mm/s. The strain state was kept for 60 s, and the data for force change were noted. The probe was then raised to its normal height, which was 30 mm above the surface. Force data were used to calculate the %SR of each OG sample using eq 4:
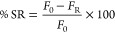
4where *F*_0_ is the maximum force in the SR curve and *F*_R_ is the residual force.

### Drug Release Studies

The release of curcumin from the OGs was evaluated in a tablet dissolution apparatus (DS-8000, Lab India analytical Instruments Pvt. Ltd., Maharashtra, India). The dissolution apparatus type-I (basket) was used for this study. The dissolution vessels were filled with 400 mL of phosphate buffer solution (PBS) containing 0.25% w/v sodium lauryl sulphate (pH 6.8, 37 °C). 1.0 g of the OGs was kept in the basket. The curcumin release study was conducted for 3 h. The rotation of the basket was fixed at a speed of 100 rpm. Then, at a specified time period (5, 15, 30, 45, 60, 90, 120, 150, and 180 min), 5 mL of the dissolution media was withdrawn and the same volume was replaced with PBS. The samples were analyzed for curcumin content at the wavelength of 430 nm in a UV–visible spectrometer (UV-1900i, Shimadzu, Japan). The study was done in triplicate.

### Ex Vivo Intestinal Permeation Studies

**Figure 1:**
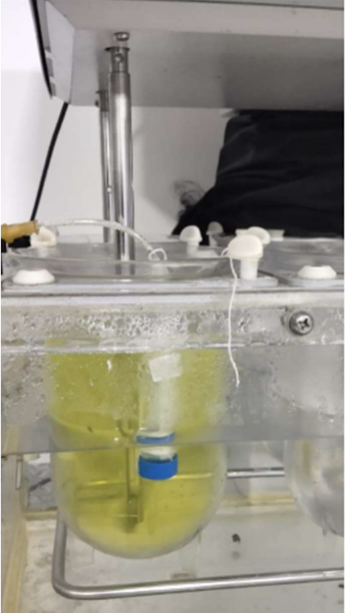
Experimental setup for ex vivo intestinal permeation studies.

The permeation of curcumin through everted goat intestine was evaluated in a tablet dissolution apparatus (DS-8000, Lab India analytical Instruments Pvt. Ltd., Maharashtra, India) following the previous procedure with slight modification.^35^ The dissolution apparatus type-II (paddle) was used for this study. The dissolution vessels were filled with 900 mL of releasing medium made with PBS containing 0.25% w/v sodium lauryl sulphate (pH 6.8, 37 °C).^35^ A 15 mL centrifuge tube was taken and a small piece of 3 cm length and 1.5 cm width was separated from the tube. A hole was created in the bottom part of the tube for the withdrawal of the sample. Fresh goat intestine was collected from the local slaughter house at 4 °C in normal saline medium. 5 cm long intestine part was sliced and washed properly with normal saline at 4 °C to remove all food residues. The intestine was everted and fitted to the tube to cover the broken part of the tube. Further, the intestine was tied tightly at both ends with the help of a thread. 10 mL of releasing medium was filled into the tube and fixed in the dissolution vessel as shown in Figure 1. 1.0 g of OG 17.5 formulation was chosen for the permeability study and was kept in the vessel. The curcumin permeation study was conducted for 3 h by rotation of the paddle fixed at a speed of 100 rpm. The curcumin permeation through the intestine was measured by withdrawing 5 mL of sample from the tube at 60, 120, and 180 min. The samples were analyzed for curcumin content at the wavelength of 430 nm in a UV–visible spectrometer (UV-1900i, Shimadzu, Japan). The study was done in triplicate.

## Results and Discussion

### Preparation of OGs

The use of edible OGs derived from healthy oil and wax in food applications, especially for oral delivery of a drug, is a growing field of research.^36^ In OGs, the organic liquid is trapped in a three-dimensional thermo-reversible network in which the oleogelator plays a key role.^3^ Edible oil like WGO is rich in tocopherols, carotenoids, sterols, and steryl ferulates and is reported to have several beneficial effects on health.^7^ Thus, oil from wheat germ may be utilized as a source of essential components in our daily food menu to promote good health.

**Figure 2:**
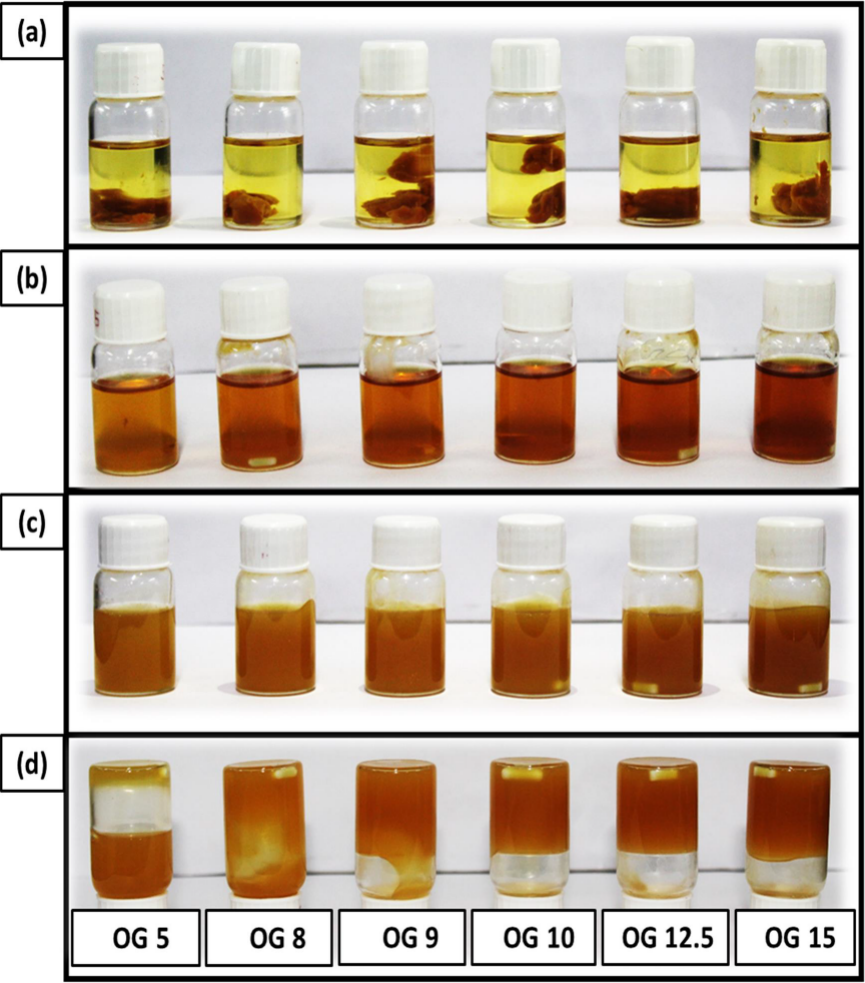
Pictures of the prepared OGs, OG 5, OG 8, OG 9, OG 10, OG 12.5, and OG 15: (a) JFW in WGO after weighing; (b) after heating at 70 °C; (c) after cooling at room temperature; (d) inverted vials.

In our study, WGO underwent oleogelation in the presence of JFW, which is a very new and less explored ingredient for OG preparation. To find the CGC of the JFW- and WGO-based OGs, initially, 5% (w/w), 8% (w/w), 9% (w/w), 10% (w/w), 12.5% (w/w), and 15% (w/w) of JFW in WGO were weighed in 10 mL vials (Figure 2a). It was observed that JFW remains immiscible in WGO at room temperature (∼28 °C). As we heated the mixture to 70 °C, the solution turned to a homogeneous light brown translucent solution (Figure 2b). Further on cooling to room temperature 10% (w/w), 12.5% (w/w), and 15% (w/w) turned to opaque brown gel whereas 5% (w/w), 8% (w/w), 9% (w/w) remained in the semisolid form (Figure 2c,d). The inverted vial technique was used to affirm the formation of the OGs. It was noticed that the inversion of the vial did not cause the downward flow movement of the 10% (w/w), 12.5% (w/w), and 15% (w/w) of JFW in WGO formulations. It indicated that the CGC of the JFW in OGs is 10% (w/w).

**Figure 3:**
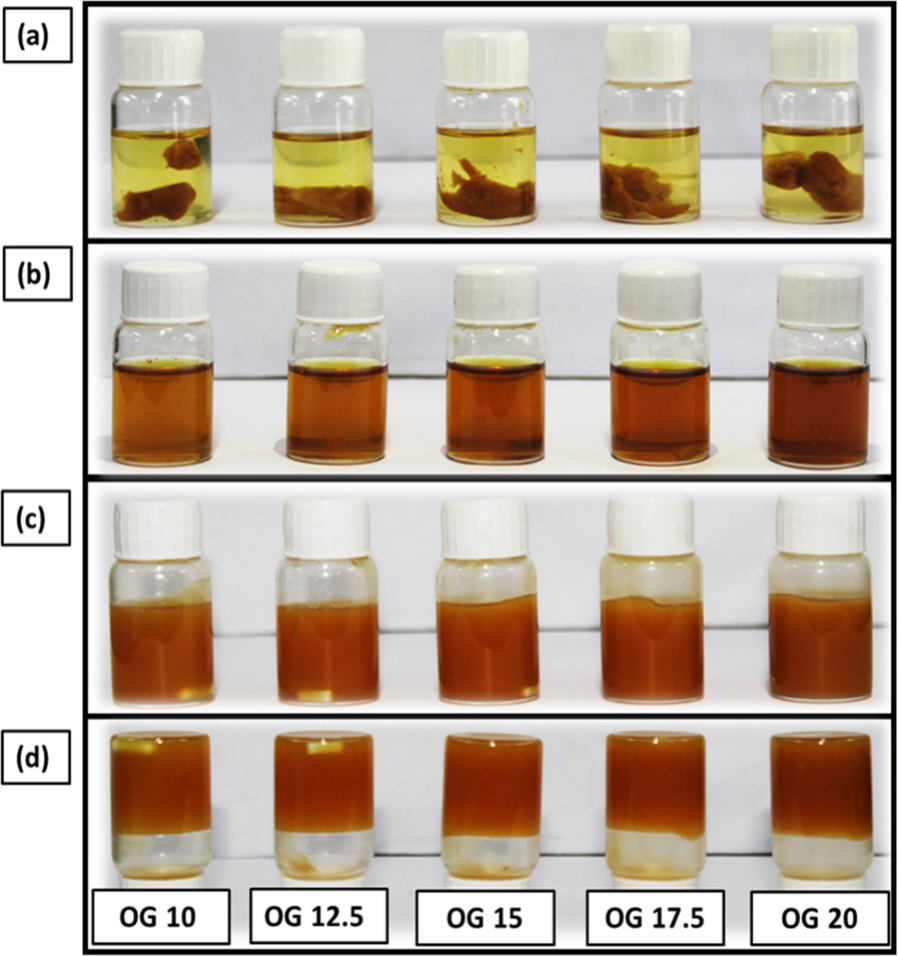
Pictures of the prepared OGs, OG 10, OG 12.5, OG 15, OG 17.5, and OG 20: (a) JFW in WGO after weighing; (b) after heating at 70 °C; (c) after cooling at room temperature; (d) inverted vials.

After confirming the CGC, further analysis was done by preparing OGs by varying the concentration of JFW in WGO from 10% (w/w) to 20% (w/w) by increasing 2.5% (w/w) for each sample. We added JFW to WGO (Figure 3a), where the JFW is insoluble and settled down. This was then heated to 70 °C in a water bath with continuous stirring by using a magnetic stirrer. This resulted in a homogenized light brown-colored clear solution (Figure 3b). Then the samples were allowed to cool at room temperature. Initially, as the temperature was lowered a cloudy mixture was formed, and as time progressed viscosity of the mixture increased and finally turned to semisolid stable OGs (Figure 3c). This process is called oleogelation. The formation of a cloudy mixture confirms the nucleation of JFW. Inverting the vial verified the formation of stable OGs (Figure 3d).

### Study of Oil Released and Oil Binding Capacity

**Figure 4:**
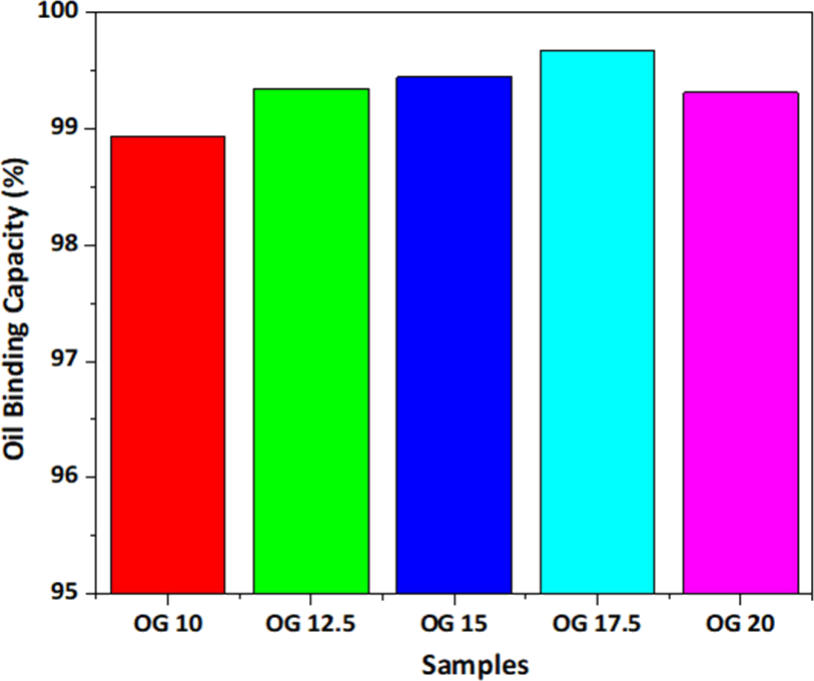
Oil-binding capacity of OGs based on different wax concentrations.

The stability of OGs is analyzed by measuring OG’s ability to bind oil in it. Thus, the stability of OGs was analyzed by calculating oil released and OBC. Higher the percentage value for OBC, the higher the stability. The OGs made from JFW were proven to be efficient in binding the oil. All the OGs showed %OBC greater than 98%. This again proves the stronger mechanical strength possessed by the OGs is due to their higher OBC value.^37^ The crystalline phase of 10% JFW concentration was proven to be the minimum wax concentration required to develop a network structure that can hold oil within. Also, there is a slight increment in OBC values from OG 10 to OG 17.5 and a decrease in value in OG 20 as in Figure 4. This result gives proof for observations obtained from the other characterization. Also, the presence of a long chain of alkanes and ester groups in the JFW may accelerate the binding capacity.^38^ Thus, synthesized OG formulations were proved to be highly stable.

### Colorimetric Analysis

**Figure 5:**
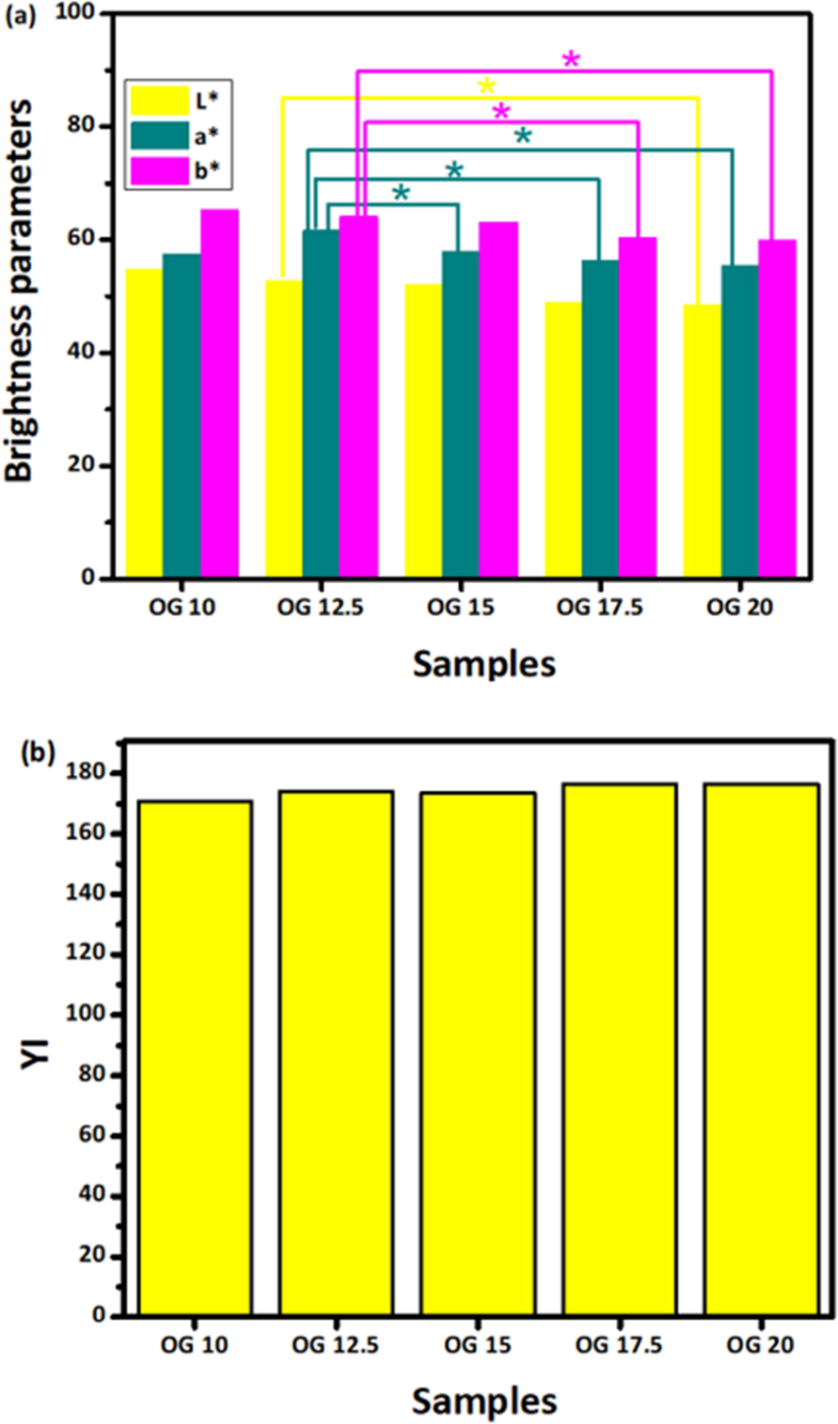
Color parameters of the OGs: (a) L* values; a* values; and b* values. (*p* < 0.05). Significantly different values are represented with the symbol “*”. (b) Graph representing YI values for different OG formulations.

The goal of this characterization is to analyze the color of the OGs using reflectance measurements under fresh conditions or during storage. L*, a*, and b* or CIE Lab are defined by Commission Internationale de l’Eclairag and are recognized widely for color measurement in food products.^39^ This theory states that all colors are a mix of red, green, and blue whose receptors are existing in the human eye.^40^ The L* value ranging from 0 to 100 is referred to as the lightness component. Since all our OG systems had shown an L* value close to 40–60, it was concluded that OGs samples are lighter in color (Figure 5a).^34^ The L* value decreases as the concentration of JFW increases in OG formulations. OG 12.5 and OG 20 show a significant difference in their values among all the other combinations giving similar results. Thus, the luminescence of the OG system reduces with respect to fat components. Moreover, a* and b* are the chromatic components ranging from green to red and blue to yellow, respectively (Figure 5a). For the values of a*, the range follows −ve (red) and +ve (green). For all the OG formulations, the a* value was found to be positive. This gives a hint about the presence of a better fraction of the red hue. OG 12.5 shows a higher a* value than OG 10. However, further addition of wax lowers a* values. Additionally, the b* value in all the formulations appeared to be positive ranging from 59–66 values. The b* values display −ve (blue) to +ve (yellow). As OG appeared to be brownish-yellow, a positive b* value indicates a significant proportion of yellow as given in other literature studies.^41^ There is a downward trend in values from OG 10 to OG 20, comparable to the behavior of a* values. A significant difference can be observed in values measured from OG 12.5 with OG 17.5 and OG 20 in the case of both a* and b*. YI calculation done as eq 3 is represented in Figure 5b. YI is the degree of yellowness, used chiefly to quantify soiling, scorching, and product degradation by chemical exposure, light, and over-processing. These types of degradation are represented with a single value. YI value increases as JFW concentration increases. The YI value in OG 15 was somewhat lower than in OG 12.5, indicating exceptional behavior. Additionally, there is no significant difference between the YI values of prepared OG formulations.

### Fourier Transform Infrared Spectroscopy

**Figure 6:**
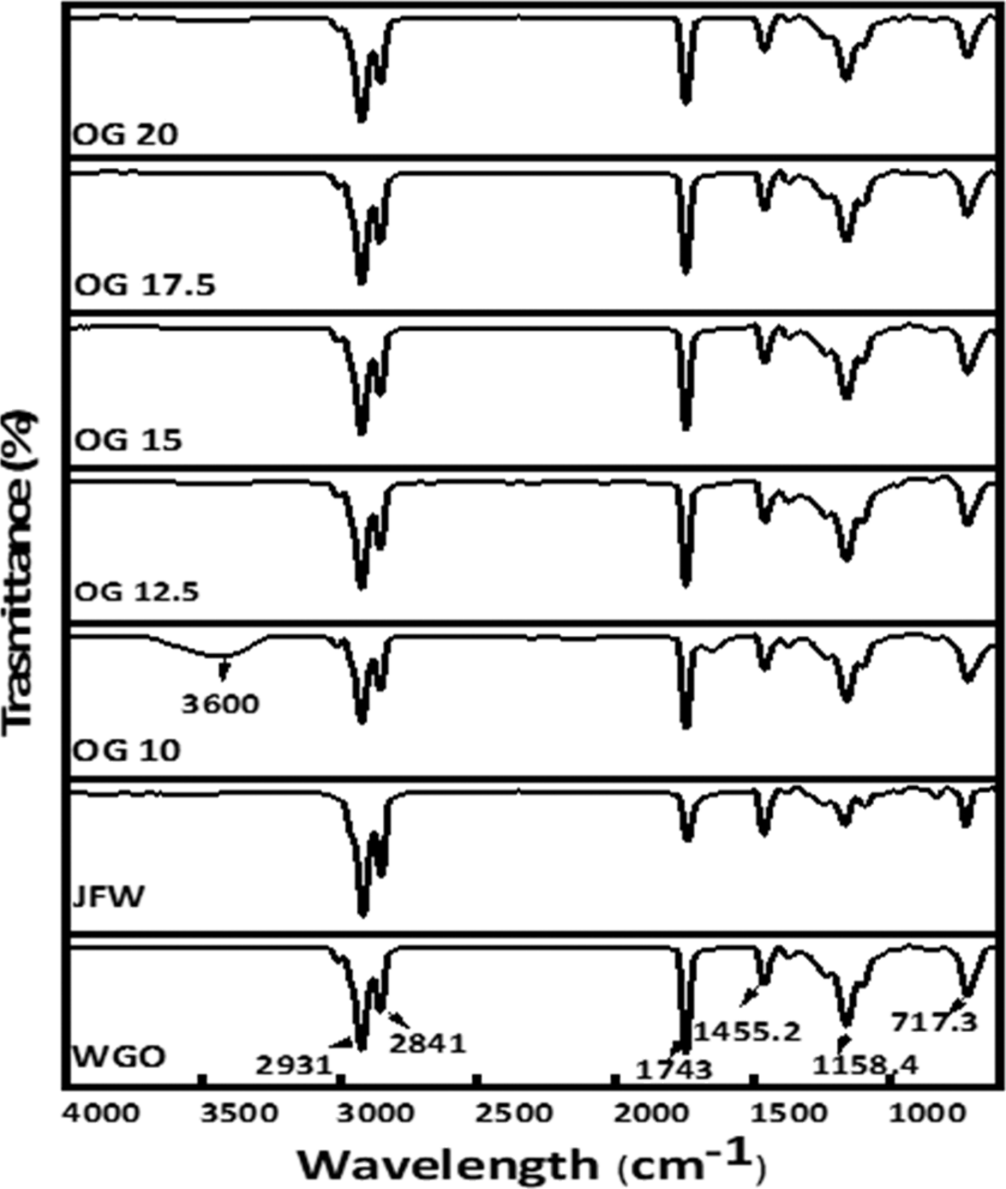
FTIR spectra of JFW, WGO, and OGs with different concentrations of JFW.

Fourier transform infrared (FTIR) spectroscopy was used to determine the presence of functional groups and chemical interactions in the prepared OG formulations.^42^ OG 10 shows a broad peak in the wavenumber range of 3700–3200 cm^–1^ different from all other formulations (Figure 6). This gives OG 10 an exceptional character from others. This property can be explained by the fact that in OG 10, hydrogen bonding is a strong reason for its gelation property, but as the concentration of JFW increases, the influence of hydrogen bonding in gelation decreases dramatically, or even disappears entirely, and other noncovalent interactions take over. The presence of noncovalent interactions causes the formation of a gelation network during the interaction of wax and oil.^43^ The FTIR spectra of JFW and WGO also exhibited almost similar characteristic IR absorption signals. The individual spectrum of both unadulterated parts exhibited a noticeable signal for carbonyl (−C=O) stretching of esters linkage characteristics of triacylglycerides. This vibrational signal was observed at 1720 and 1735 cm^–1^ in JFW and WGO, respectively. The FTIR spectrum of various compositions also shows a strong vibrational frequency in the range of 1740 to 1751 cm^–1^, which demonstrates the presence of the carbonyl group (−C=O) whereas, the dual peak in the range of 2922 to 2855 cm^–1^ shows the existence of the alkane group (C–H) from the CH_3_ and CH_2_ groups.^44^ Similar stretching vibrations were also observed for JFW and WGO. The peak at ≈1743 cm^–1^ can be identified due to the carbonyl group stretching vibrations, these peaks are also seen in oil and wax. The CH_3_ and CH_2_ groups showing C–H bending (present in both WGO and JFM) were observed at ≈1455 cm^–1^. The peaks at ≈1159 cm^–1^ can be contributed by C–O stretching vibration from the functional groups of C–O–H and C–O–C, which are present in both oil and gelator molecules. The peak in ≈720 cm^–1^ is attributed to the presence of (CH_2_)_*n*_ bending vibration. The inclusion of the aforementioned functional groups in both JFW and WGO contributed to the peak.

### XRD

**Figure 7:**
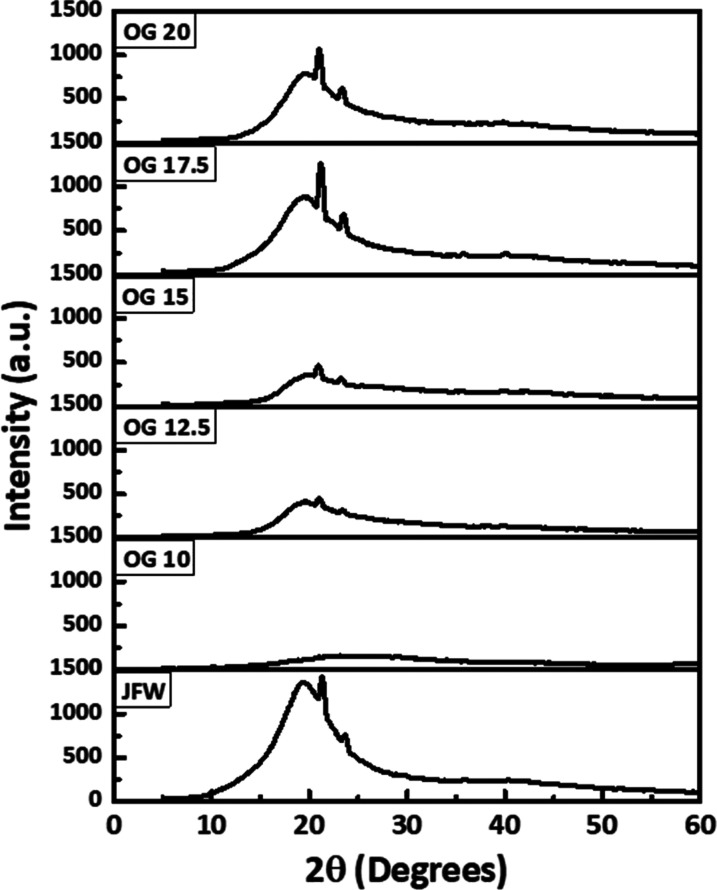
XRD spectra of JFW and OGs with different concentrations of JFW.

The diffraction patterns of JFW and WGO-based OGs were recorded using an X-ray diffractometer. The scanning was done within the range of 5 to 80° 2θ in a copper source at scanning speeds of 5° 2θ/min to get an idea about the nucleation which grew in size and resulted in the fiber structures.^45^ This influences the crystallinity of the OGs. The peaks at ≈21.38 and ≈23.67° on the XRD graph for JFW are regarded to be the typical peaks of crystallization for JFW because other waxes have comparable crystalline properties.^46^ Except OG 10, all OG XRD patterns exhibit a notable intense peak at ≈21.38 and ≈23.67° (Figure 7). This is most likely due to OG 10’s lesser amount of JFW content. As the concentration of JFW increases, the intensity of the peak in the OG formulation is also enhanced. The prepared OGs from 17.5% of JFW have better crystallization than JFW. The fact that OG 20 has a lower peak intensity than OG 17.5 might be related to crystal flaws in the system since the wax concentration is higher than that required for effective OG synthesis. The XRD peaks in the range of ≈10° 2θ to ≈30° 2θ from OG 10 to OG 20 were deconvoluted using the Gauss peak fitting function in Origin Pro software. Peak parameters like peak position and full width at half maximum (FWHM) values of the deconvoluted peaks have been tabulated which can be used to calculate the *d*-spacing, crystallite size, and lattice strain of the crystallite regions in the OGs (Table 1). These data give a clear idea about the structural changes in OG as the concentration of JFW is increased. The differences in the FWHM of observed peaks can be related to the crystalline nature of the OGs.^46^ The average value of FWHM is decreased as the JFW concentration increases in the systems until OG 17.5. This is due to the reduction of the amorphous nature of the crystal network in the OGs. However, OG 20 shows a slight increase in FWHM values as further addition of wax increased the amorphous nature of the OG. The lowest average FWHM of OG 17.5 indicates that this is comparatively more crystalline than the remaining OGs. Afterward, the mean values of d-spacing, crystallite size, and lattice strain were calculated. As we examine the average values of *d*-spacing, there is a decrease in the corresponding values as the concentration of the JFW was increased in the OG system up to 17.5. Alteration of the lattice positions is the reason for the changes in obtained values for *d*-spacing.^47^ Similarly, the average crystallite size is enhanced as the JFW concentration increased from OG 10 to OG 17.5, but a decrease in the value of OG 20. This again proves OG 17.5 is the best OG formulation among the five different formulations. The same result we can correlate with microscopic images, as well as OG 17.5, gives a denser fiber-like structure. Further, the mean values of lattice strain were found to be decreased as the concentration of the JFW was increased up to OG 17.5. OG 20 shows a slight increase in lattice strain which can be due to the increase in crystal defect as the JFW exceeds the concentration needed for OG formation.^4^ However, up to OG 17.5, the crystal defects gradually decrease as the JFW concentration in OGs increases.

**Table 1:** XRD Parameters Obtained from Deconvoluted Peaks

oleogels	peak	peak position (°2θ)	FWHM (°2θ)	*d*-spacing (Å)	crystallite size (nm)	lattice strain
OG 10	1	11.17	1.27	8.01	6.57	0.06
	2	20.48	6.89	4.39	1.22	0.17
	3	25.88	14.87	3.48	0.57	0.28
	average		7.68	5.29	2.29	0.17
OG 12.5	1	18.93	3.87	4.74	2.17	0.1013
	2	21.05	0.36	4.27	23.45	0.0085
	3	21.53	7.89	4.18	1.07	0.1811
	4	28.98	7.84	3.12	1.09	0.1324
	average		4.99	4.08	6.94	0.11
OG 15	1	19.67	4.67	4.57	1.8	0.12
	2	20.94	0.36	4.29	23.45	0.01
	3	23.28	0.44	3.87	19.26	0.01
	4	25.12	9.73	3.59	0.87	0.19
	average		3.80	4.08	11.35	0.08
OG 17.5	1	19.46	6.46	4.62	1.30	0.16
	2	21.19	0.36	4.25	23.46	0.01
	3	23.52	0.42	4.25	20.19	0.01
	4	26.04	5.86	3.46	1.45	0.11
	average		3.27	4.14	11.60	0.07
OG 20	1	19.54	5.09	4.60	1.65	0.13
	2	21.02	0.37	4.28	22.82	0.01
	3	23.37	0.45	3.85	18.84	0.01
	4	23.47	12.23	3.84	0.69	0.26
	average		4.53	4.14	11.00	0.10

### Microstructure Studies

**Figure 8:**
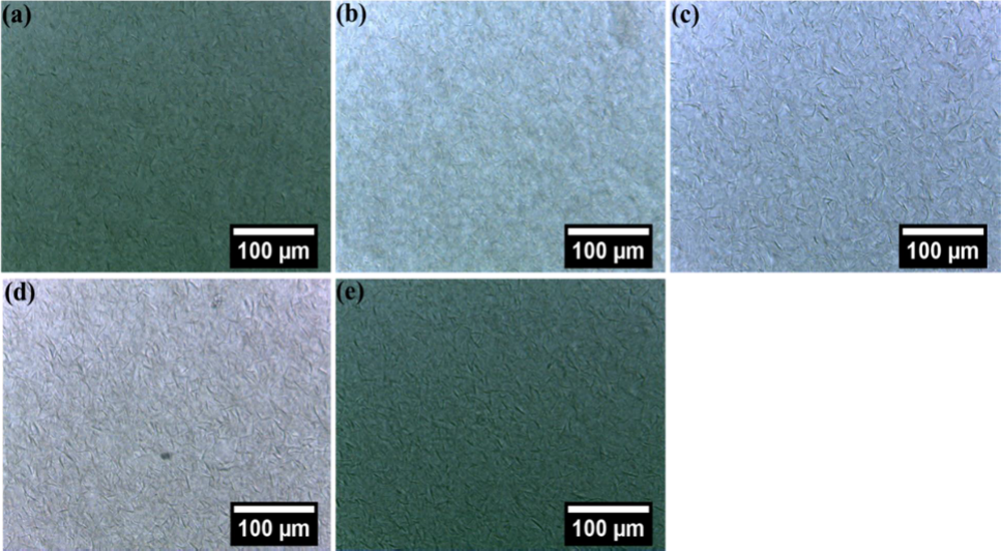
Bright-field micrographs of the prepared OGs: (a) OG 10, (b) OG 12.5, (c) OG 15, (d) OG 17.5 and (e) OG 20.

**Figure 9:**
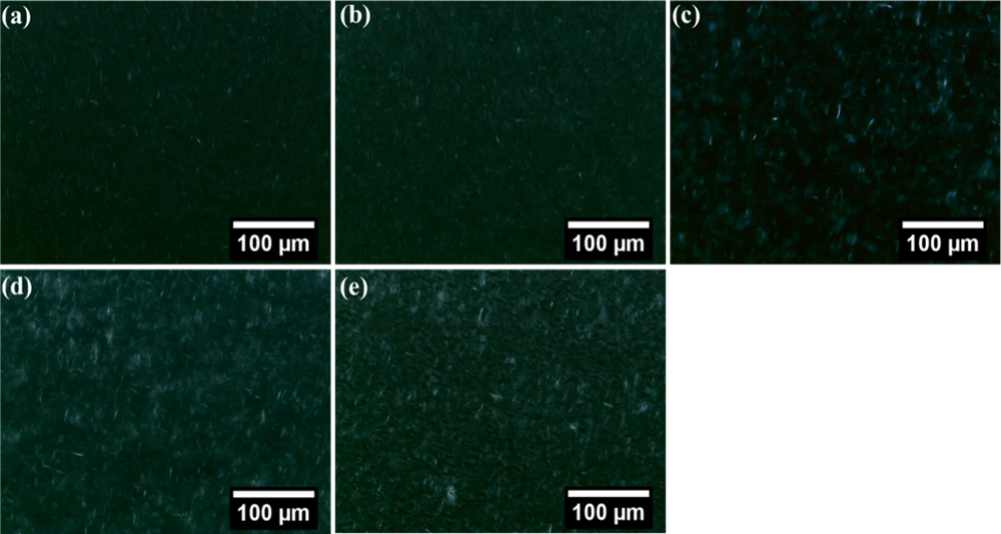
Polarized light micrographs of oleogel formulations: (a) OG 10, (b) OG 12.5, (c) OG 15, (d) OG 17.5 and (e) OG 20.

Optical microscopy was used to study the morphology of crystals formed in OGs prepared using JFW and WGO.^48^ Micrographs of the OGs taken with bright field and polarized light microscopes were utilized to visualize the fat network generated in the OGs. The physical property of the OGs can be identified by analyzing the polymorphism and morphology as the OGs based on wax relies on the entrapment of the oil phase through wax crystals. The bright-field microscopic images suggested the formation of fiber structures which are formed through the entanglement of crystals similar to previous works (Figure 8).^49,50^ This fibrous network appearance can be due to the high content of wax esters in JFW. Analysis of the polarized light micrographs of OG revealed that spherulites or clustered crystallites were formed in OGs prepared by JFW and WGO (Figure 9).^51^ The spherulite-like construction was shaped because of the aggregation of needle-like crystals, which were grown from a nucleation center, and also revealed the formation of a network-like structure. Moreover, with the increase of the oleogelator (JFW) content, an increase in the crystallite size was observed. Interestingly, polarized light micrographs of OG 17.5 demonstrated the formation of larger crystallites among the OGs investigated in the present study. Molecular self-assembly of the gelator molecule including hydrogen bonding, van der Waals forces, π–π stacking, and other hydrophobic interactions gives the waxes or any LMGO a three-dimensional fibrous network. The molecular units of waxes are primarily linear. The fibrous bundles found in OG 10, OG 12.5, and OG 15 were relatively shorter. Interestingly in OG 17.5, compared to other OG systems, longer and denser fiber bundles were seen. Generally, an increase in JFW concentration is the reason for the denser network structure.^52^ Further, the interconnectivity of the fiber bundles was also increased as the JFW concentration was increased. A similar finding has been reported in palmitic acid OG and stearic acid OG.^53^ Analysis of the polarized light micrographs proposed the presence of the oil phase within the network structure of the gelator fibers. As the concentration of the JFW in the OGs increases, there is a rise in the fiber length and the fiber–fiber junctions.

### Metallurgical Microscopy

**Figure 10:**
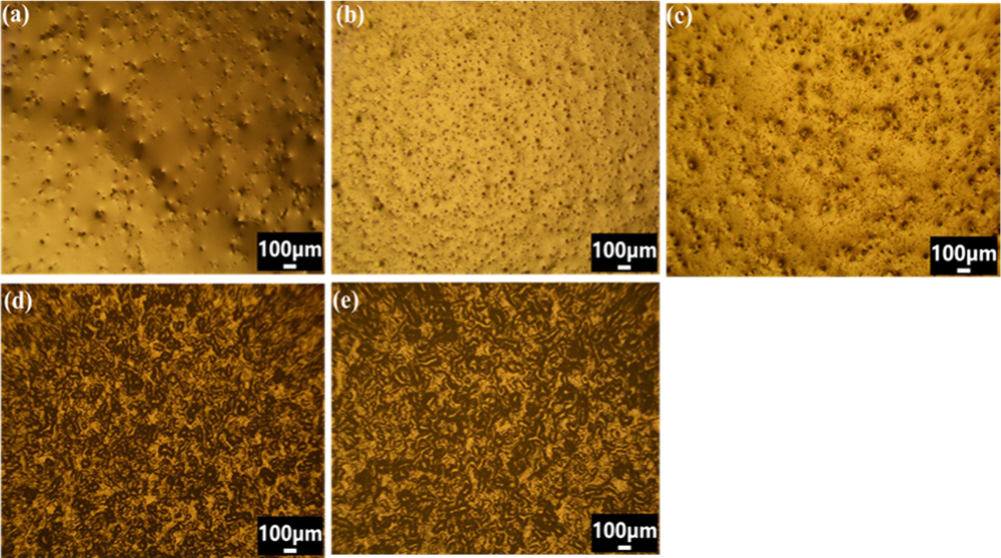
Metallurgical micrographs of the prepared OGs: (a) OG 10, (b) OG 12.5, (c) OG 15, (d) OG 17.5, and (e) OG 20.

Metallurgical studies were done in order to analyze the topology of the prepared OGs. Images of OG 10 to OG 20 were taken for comparing their topological differences as the wax concentration is varied (Figure 10). OG 10 to OG 15 shows a very smooth surface having nonuniform patches on the surface. Darker patches observed are globular structures formed due to the gelation of oil by wax. As observed, the wax concentration enhances the globular formations on the OGs. However, in OG 17.5 and OG 20, the smooth surface is gone and as a result, we can observe a huge difference in their images with other OGs. In the case of OG 17.5 and OG 20, very large globular agglomerates are formed unevenly on the surface. Thus, we can conclude as the concentration of wax increases in the prepared OG formulations, and globular structure formation is also enhanced.

### Mechanical Study

**Figure 11:**
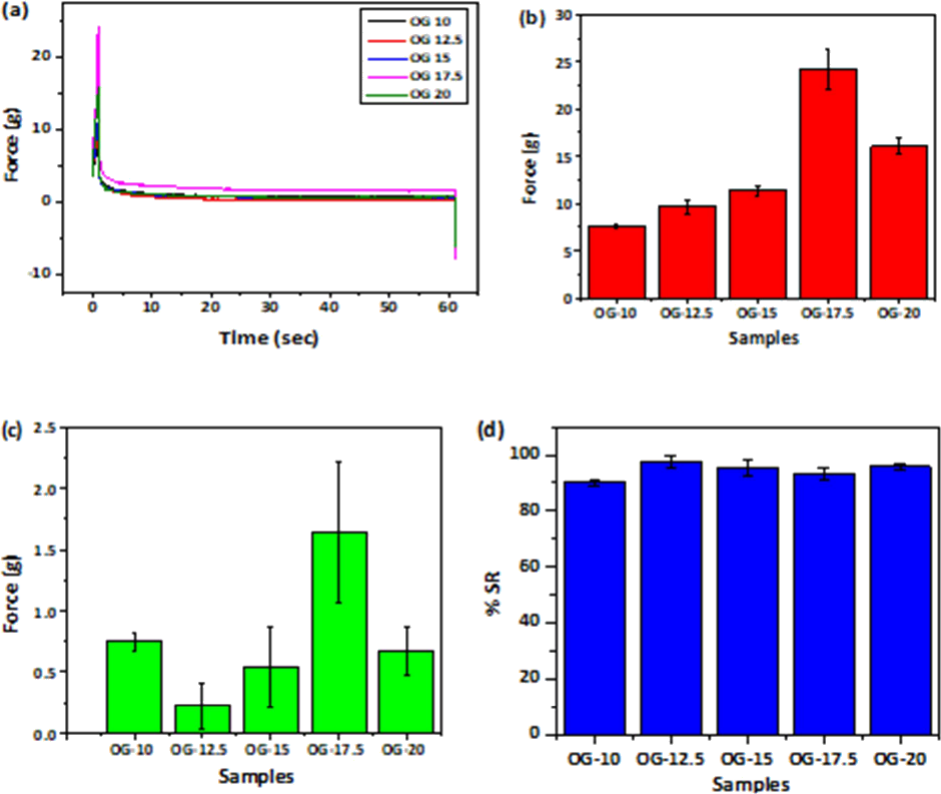
Mechanical properties of the OGs, (a) SR profile, (b) *F*_0_ values, (c) residual elastic force in OGs, and (d) %SR values.

SR studies of the OGs were analyzed using mechanical parameters (Figure 11). Macroscopic level changes in the gel structure can be analyzed using this technique as proven in previous works.^4^ Hence, this characterization aimed to understand the macroscopic changes in the gel as the concentration of JFW is increased. The stress release profiles were studied from the viscoelastic properties of the prepared OGs (Figure 11a). As the OG is externally distorted, stress is exerted on the probe due to the wax network and the fluid pressure of the entrapped oil together, which is demonstrated in the SR profile.^53^ The steadiness of the OG systems is projected using the maximum attained force (*F*_0_) (Figure 11b). There is a reduction in force values with time as the strained condition was retained for 60 s, after reaching the maximum force. The maximum force achieved just after the compression stage (*f*_max_), was increased with the consequent increase in the JFW concentration up to OG 17.5, but a decrease in value is observed in OG 20. Subsequently, during the relaxation stage, the force was exponentially decayed until the force value leveled to a minimum constant value (*f*_min_).^54^ In this case, too, similar behavior by OG 20 and for rest formulations it was found that there was an increase in the force values with the corresponding increase in the concentration of JFW. Alteration in the mechanical properties of the OGs with the alteration in the composition is a well-documented phenomenon.^53^ The overall effect of molecular rearrangement in the OG component especially the gelator molecules result in the degeneration of force. Destruction of the gelator fiber structure and the network assemblies can be a major reason for this. The residual force remaining after the completion of the analysis represents the residual elastic component remaining within the OGs (Figure 11c).^54^ The decay profiles of the force were dependent on the composition of the OGs. As the JFW concentration in OG increases, systems become more crystalline and this can cause the breakage of the network structure.^53^ The percentage of SR (%SR) was calculated as per the equation given in eq 4. The %SR provides evidence about the ability of the sample to absorb the energy associated with the induced strain (Figure 11d). As expected, with the increase in the concentration of JFW, there was a corresponding decrease in the %SR values from OG 12.5 to OG 17.5. The slight increase in SR% in OG 20 can be due to the crystal defects present in the system which supports the similar exceptional behavior shown in XRD and optical analysis. It revealed that when wax concentrations increase, the stress release value decreases and noncovalent interactions in the OG emerge. The newly forming interactions are capable of storing the forces without releasing them.

### Crystallization Studies

**Figure 12:**
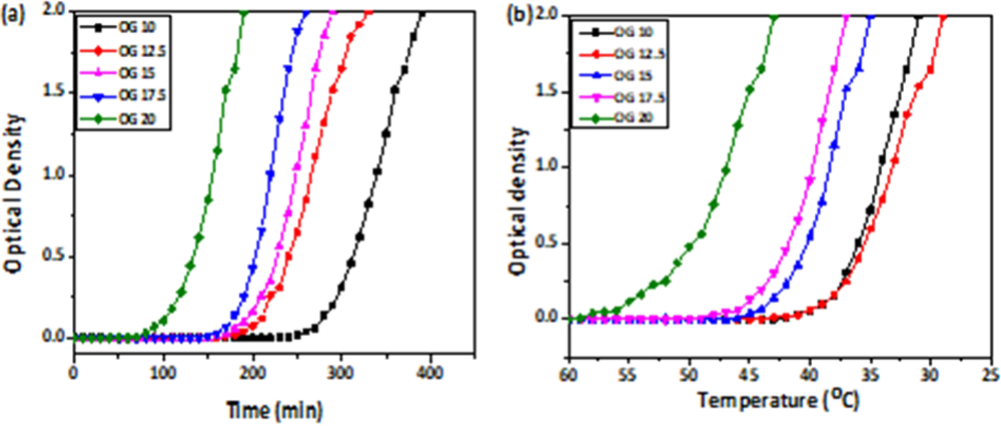
Gelation kinetics of all the formulations (a) w.r.t. time (min), (b) w.r.t. temperature (°C).

Crystallization of OGs is the process of controlling the mobility of the oil, which is a triacylglycerol, using a gelator system and organizing them into a compact structure. This is majorly due to the noncovalent interactions between the triacylglycerol, thus resulting in immobilization.^55^ It is critical to understand the gelation behavior of fats when using OGs for industrial applications. Here, the measurement of gelation is done with respect to OD versus both time and temperature. Three mechanisms of gelation like nucleation, crystal growth, and saturation can be observed.^56^ In the given graph, the saturation phase is not represented as the OD exceeded 2, and thus further measurement was not possible (Figure 12). Gelation over time (Figure 12a) demonstrates that when the concentration of JFW increases from OG 10 to OG 20, the time necessary to initiate gelation increases as well. OG 20 with a higher wax concentration starts the gelation at 80 min after keeping the prepared formulation at room temperature. On the other hand, OG 10 shows gelation after 280 min. This shows the significant role of gelator molecules in the oleogelation process. The change in OD of the OGs recorded in this study is shown in Figure 12b as a function of temperature change. The OG formulations are maintained for cooling once they have been melted. OD is assessed throughout the cooling process. OG 20 can form a gel as soon as it reaches 55 °C. The temperature for gelation drops as JFW rises in the system.^56^ OG 12.5 and OG 10 give the same gelation temperature at 37 °C. Nevertheless, OG 10 shows a sharp increase in OD when compared to OG 12.5 (Figure 12b). As a result, increasing the amount of gelator molecules in OGs improves the physical or chemical interaction inside triacylglycerols, resulting in more crystalline OGs. The intentional behavior of OG 12.5 on the time scale and OG 15 on the temperature scale cannot be explained at this stage. In further studies, we can analyze this extraordinary behavior.

### Drug Release Studies

**Figure 13:**
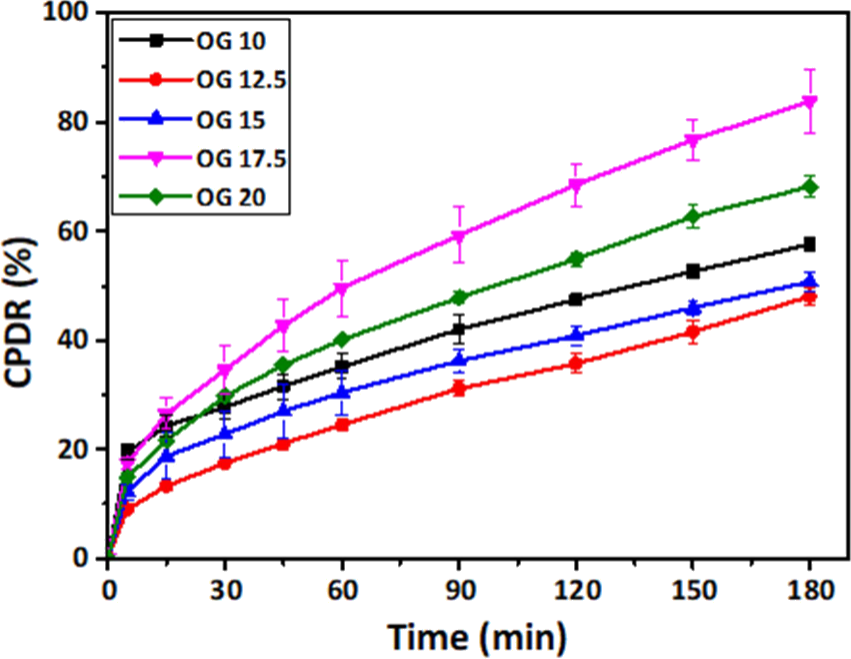
Drug release profile of OGs.

Curcumin, the principal curcuminoid of the golden spice turmeric, has a wide range of biological effects including anti-inflammatory, anti-microbial, antioxidant, anti-cancer, and many more.^57^ As a result, it has the potential to be used as herbal medicine. Curcumin’s low water solubility is the most significant impediment to its absorption into our bodies.^51^ Additionally, it is crucial to develop a carrier for curcumin that is nontoxic, and edible for oral administration of curcumin. The bioavailability of the carrier systems as they should act at the site of action causing the release of encapsulated curcumin is also important.^58^ This release was calculated as cumulative percentage drug release (CPDR) (Figure 13).

The experiment was conducted for up to 180 min. It is observed that %CPDR is less for OG 12.5 followed by OG 15. As hydrogen bonding dominates in OG 10, there is a chance of higher encapsulation of curcumin in it causing the high CPDR% when compared to OG 12.5 and OG 15. In line with other characteristics, OG 17.5 has a greater CPDR% than OG 20. Due to crystal defects, OG 20 is unable to absorb curcumin. This might be one of the reasons for the lower CDPR value.

The curcumin release profiles were fitted to Korsmeyer–Peppas (eq 5), kinetic models for drug release.^59^ The least-squares fit method was used for the fitting, and the results are in great agreement with the model (*R*_2_ > 0.99).^5^Table 2 consists of the values of parameters obtained from the Korsmeyer–Peppas model. The diffusion constant (*K*) represents the rate of drug diffusion. It was evident that the rate of diffusion of curcumin molecules increased significantly as the JFW proportion increased in OGs. The diffusion exponent (*n*) represents the type of drug release mechanism. If the “*n*” value is ≤0.45, the release is mainly mediated by Fickian diffusion.^5^ Whereas, if the “*n*” value is in the range of 0.5 and 0.89, the release is occurring via non-Fickian transport, while the polymer swelling mediated diffusion process happens if the “*n*” value is ≥0.89. In our study, the “*n*” values of the OG formulations are less than 0.45. Thus, Fickian diffusion-mediated drug release was detected in all of our carrier systems.
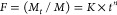
5where *F* is the amount of drug released, *M_t_*/*M* is the fraction of drug accumulated in the solution at time *t*, *K* is the kinetic constant, and *n* is the diffusion exponent.^5^

**Table 2:** Korsmeyer–Peppas Model Parameters of Curcumin Release from the OGs

formulations	*K*	*n*	*R*_2_
OG 10	0.840 ± 0.112	0.382 ± 0.020	0.997 ± 1.231
OG 12.5	0.917 ± 0.145	0.380 ± 0.034	0.988 ± 0.008
OG 15	1.163 ± 0.105	0.340 ± 0.016	0.990 ± 0.002
OG 17.5	1.442 ± 0.032	0.305 ± 0.003	0.975 ± 0.016
OG 20	3.503 ± 0.296	0.192 ± 0.012	0.998 ± 0.002

### Ex Vivo Intestinal Permeation Studies

**Figure 14:**
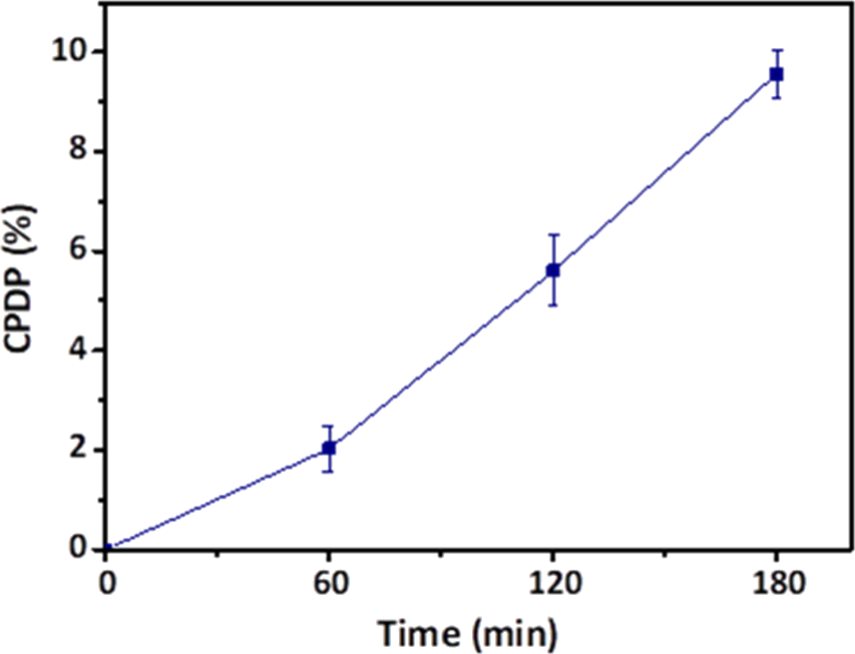
Ex vivo intestinal permeation profile of curcumin (OG 17.5).

The permeation study was conducted by using goat intestine by following the procedure as shown in Figure 1. The OG 17.5, which showed the highest drug release was selected for the permeability analysis. The ex vivo intestinal cumulative percent drug permeated (CPDP) of curcumin from OG 17.5 was observed to be 9.56 ± 0.50% per cm^2^ after 3 h of analysis (Figure 14, Table 3).

**Table 3:** CPDP of Curcumin from OG 17.5 at Different Time Intervals

time (min)	CPDP (%) per cm^2^
0	0 ± 0.00
60	2.02 ± 0.45
120	5.60 ± 0.71
180	9.56 ± 0.50

In order to predict and correlate the in vitro curcumin release observed already with the CPDP values obtained in this study, it is required to fit the CPDP values into a proper mathematical model. Thereby, the CPDR of OG 17.5 from in vitro analysis was fitted to zero order, first order, Higuchi order, and Korsmeyer–Peppas mathematical models (Table 4). By comparing the correlation coefficients of all models, the CPDP was best fitted to the Korsmeyer–Peppas mathematical model (*R*_2_ = 0.99). The diffusion exponent (*n*) value was found as 1.38, which indicated the drug permeation followed the supercase II transport.^35^

**Table 4:** CPDR of OG 17.5 from in Vitro Analysis Fitted to Different Mathematical Models

parameters	zero order		first order		Higuchi		KP model	
	*K*_0_	*R*^2^	*K*_1_	*R*^2^	*K*_h_	*R*^2^	*n*	*R*^2^
	0.05	0.97	0.00	0.97	0.57	0.82	1.38	0.99

## Conclusions

In summary, OGs derived from JFW and WGO were synthesized, characterized, and analyzed for the oral delivery of curcumin. Initially, to determine the CGC, OG formulations from 5 to 15% (w/w) of JFW in WGO oil were prepared. The CGC of JFW to induce the gelation of WGO was found to be 10% (w/w). Among the OGs, OG 10 showed hydrogen bonding, whereas, other OGs lack the peak corresponding to hydrogen bonding. This can be explained as the domination of other noncovalent interactions other than hydrogen bonds in the OGs for gelation. Gelation kinetics and XRD confirmed that the formation of OGs was accompanied by the coupling of heterogeneous nucleation with one-dimensional growth of the gelator fibers. The microscopic studies of OGs mostly contained the crystal network structure of fiber-like fat crystals. OG 17.5 was shown to have denser fibers and may be called the greatest OG system among the others. Metallurgical studies confirm the presence of large globular agglomerates on the surface of OGs as the wax concentration increases. It was also confirmed that when stress was applied, the percentage of stress release decreased as the wax percentage increased, this may be due to the formation of new bonds in the physical OGs. The curcumin-loaded OGs showed that OG 10 has a better likelihood of curcumin encapsulation when compared with OG 12.5 and OG 15, whereas, OG 17.5 has a higher %CPDR than OG 20. An increase in the JFW concentration resulted in the increased partitioning of the curcumin into the formulation. This affects the drug release and it comes in terms with Korsmeyer–Peppas model parameters of curcumin release. OG 17.5 was also tested for ex-vivo drug release into the goat intestine and it satisfied the in vitro release results. These findings might be relevant for the application of OGs in the food and pharmaceutical industries for loading and delivering bioactive components.
